# Therapeutic Mechanisms of *Vernonia amygdalina* Delile in the Treatment of Prostate Cancer

**DOI:** 10.3390/molecules22101594

**Published:** 2017-09-22

**Authors:** William Johnson, Paul B. Tchounwou, Clement G. Yedjou

**Affiliations:** Natural Chemotherapeutics Research Laboratory, NIH-RCMI Center for Environmental Health College of Science, Engineering and Technology, Jackson State University, 1400 Lynch Street, P.O. Box 18540, Jackson, MS 39217, USA; willkjohnson@gmail.com

**Keywords:** *Vernonia amygdalina* Delile, PC-3 cells, oxidative stress, DNA damage, apoptosis, necrosis

## Abstract

Prostate cancer patients have been suffering from limited treatment options due to late diagnosis, poor drug tolerance, and multi-drug resistance to almost all the current drug treatments. Therefore, it is important to seek a new alternative therapeutic medicine that can effectively prevent the disease and even eradicate the progression and metastasis of prostate cancer. *Vernonia amygdalina* Delile (VAD) is a common edible vegetable in Cameroon that has been used as a traditional medicine for some human diseases. However, to the best of our knowledge, no previous reports have explored its therapeutic efficacy against human prostate cancer. The objective of the present study was to assess the anticancer activities of VAD methanolic extracts in the prevention and treatment of prostate cancer using human androgen-independent prostate cancer (PC-3) cells as a test model. To achieve our objective, PC-3 cells were treated with various doses of VAD for 48 h. Data generated from the trypan blue test and MTT assay demonstrated that VAD extracts exhibited significant growth-inhibitory effects on PC-3 cells. Collectively, we established for the first time the antiproliferative effects of VAD on PC-3 cells, with an IC_50_ value of about 196.6 µg/mL. Further experiments, including cell morphology, lipid peroxidation and comet assays, and apoptosis analysis showed that VAD caused growth-inhibitory effects on PC-3 cells through the induction of cell growth arrest, DNA damage, apoptosis, and necrosis in vitro and may provide protection from oxidative stress diseases as a result of its high antioxidant content. These results provide useful data on the anticancer activities of VAD for prostate cancer and demonstrate the novel possibilities of this medicinal plant for developing prostate cancer therapies.

## 1. Introduction

Prostate cancer is the most common noncutaneous cancer and second leading cause of cancer-related death in North American males [1]. Men with early prostate cancer often have no symptoms or similar symptoms to diseases such as prostatic hyperplasia, and undergo no therapy. The cancer may metastasize from the prostate to other parts of the body, particularly to the bones and lymph nodes [2,3,4]. Worldwide, prostate cancer-related deaths represent the sixth leading cause of death among men [5,6]. Although cancer therapy in the form of surgery or radiotherapy is effective when the disease is detected early, many cancers are still diagnosed when cells from a primary tumor have already metastasized to other parts of the body. The main form of treatment at this point is chemotherapy, which consists of delivering drugs systemically to kill the tumor cells. However, several chemotherapeutic agents or drugs cause severe side effects in patients with prostate cancer [7,8]. The low efficacy of chemotherapy in patients with advanced cancers is reflected by the low 5 year survival rates observed in many patients with cancer [1].

Even when prostate cancer is treated, it is more likely to return, especially in the first few years after treatment. As such, this is a patient population that could benefit greatly from a natural medicinal plant that is relatively inexpensive, nontoxic, and virtually without side effects. Natural products, especially fruits and vegetables, have received increasing attention as chemopreventive agents because of their antioxidative and anti-inflammatory activities and low toxicity [9]. Recent epidemiological studies have indicated that the regular intake of a high-fiber, low-fat diet accompanied by significant consumption of fruits and vegetables significantly decreased the overall cancer risks [10,11]. The use of complementary and alternative medicine (CAM) is increasing rapidly. For example, prior studies suggested that the number of patients who visited providers or health professional of CAM exceeded the number of patients who visited primary care physicians in the United States [12,13]. Several scientific studies reported that large numbers of people with cancer are more likely to use CAM therapies as part of the disease management and rely on CAM to increase their quality of life [14,15,16].

According to the most recent statistics from the World Health Organization (WHO), herbal and plant-derived medicines are the most frequently used therapies worldwide; nearly 80% of the population in developing countries depend on these natural remedies to maintain healthcare, and there has been a 38% increase in usage in the United States within the last decade of the 20th century alone [17]. Thus, the search for more natural agents has emerged for scientists in recent years, to improve cancer prognosis and ameliorate the harmful side effects associated with chemotherapies. *Vernonia amygdalina* Delile (VAD) is an edible vegetable and a valuable medicinal plant that is widespread in Cameroon, Central West Africa. A recent study indicated that an aqueous extract of VAD ameliorated testosterone-induced benign prostatic hyperplasia in Wistar rats [18]. Previously, Izevbigie (2003) reported that some peptides (edotides) from the aqueous extract of *V. amygdalina* showed cell growth-inhibitory effects in the prostate cancer cell line, but no effect on normal human peripheral blood mononuclear cells [19].

Previously published data in our lab showed that in vitro *V. amygdalina* treatment reduced cellular viability and induced DNA damage leading to apoptosis accompanied by secondary necrotic cell death in MCF-7 cells [20,21]. Other studies indicated that *V. amygdalina* inhibits the proliferation of estrogen receptor-positive (ER+) human breast carcinoma cells in vitro [19]. In experimental animals, *V. amygdalina* showed both hypoglycemic and hypolipidaemic properties and could be used to manage diabetes mellitus [22,23]. VAD plant is very popular in Cameroon; it is a locally eaten vegetable and/or is used as traditional medicine. Even though herbal preparations of VAD are widely used for nutritional and medicinal purposes in Cameroon, there is very little scientific evidence available to support its medicinal claims. Hence, the present research was designed to use PC-3 tumor cells as a test model to assess the therapeutic efficacy of VAD leaf extracts in the management of prostate cancer.

## 2. Materials and Methods

### 2.1. Chemicals and Media

Cell culture plates and flasks (T-75 mm) were obtained from Corning Incorporated (Corning, NY, USA); Kaighn’s modification of Ham’s F-12K medium was purchased from ATCC (Manassas, VA, USA). Fetal bovine serum (FBS), and penicillin/streptomycin antibiotic solution were both purchased from Sigma-Aldrich, Inc. (St. Louis, MO, USA). PBS, trypsin, trypsin neutralizing solution (TNS), and trypan blue cell viability dye were purchased from Lonza Inc. (Walkersville, MD, USA). The 0.45 and 0.22 µm filter units (with syringe) were purchased from Millipore (Carrigtwohill, Co. Cork, Ireland). The lipid peroxidation kit was purchased from AbCam. The [3-(4,5-dimethyl-2-yl)-5-(3-carboxymethoxyphenyl)-2-(4-sulfophenyl)-2*H*-tetrazolium] (MTS assay kit was purchased from Promega Corp (specifically the CellTiter 96 AQ_ueous_ One Solution Cell Proliferation Assay; Madison, WI, USA). The comet assay and annexin V assay kits were both purchased from Trevigen Inc. (Gaithersburg, MD, USA).

### 2.2. Vernonia amygdalina Delile Preparation 

VAD leaves (4–5 kg) were collected in Bangou, West Cameroon. They were rinsed with distilled water and dried under the sun. Briefly, 100 g of dried leaves were added to 1200 mL of methanol. The mixture was heated at 50 °C for 6 h. The mixture was filtered with cheesecloth and later with Whatman No. 1 filter paper to obtain a homogenous filtrate. Excess solvents were trapped, collected and removed from the filtrate using a rotary evaporator. The extracts were then refrigerated at 4 °C until use. The preparation was performed in the Department of Chemistry and Biochemistry at Jackson State University.

### 2.3. Tissue/Cell Culture

Androgen-independent human prostate tumor cell line PC-3 was purchased from American Type Culture Collection (Manassas, VA, USA). The PC-3 carcinoma cells were cultured in Kaighn’s modification of Ham’s F-12K medium, supplemented with 10% FBS and 0.1% penicillin/streptomycin solution (Sigma-Aldrich, Inc., St. Louis, MO, USA), and grown in an incubator at 37 °C in 5% CO_2_. Fresh medium was supplemented every 48 h.

### 2.4. Cell Treatment and Determination Cell Viability

The effect of VAD on the viability of PC-3 carcinoma cells was determined by a trypan blue dye exclusion assay. Briefly, 900 μL aliquots in three replicates of the cell suspension (5 × 10^5^ cells/mL) were seeded to 12-well polystyrene tissue culture plates; 100 μL aliquots of stock solutions of VAD were added to each well using distilled water as the solvent to make up final concentrations of 125, 250, and 500 μg/mL of VAD, respectively. The control cells received 100 μL of distilled water. The cells were placed in a humidified 5% CO_2_ incubator at 37 °C for 48 h. After incubation for indicated time points, the cells were collected, an aliquot of the cell suspension was mixed with an equal volume of trypan blue, and the cells were counted under the microscope.

### 2.5. Measurement of Malondialdehyde (MDA) Level by Lipid Peroxidation Assay

The level of lipid peroxidation in VAD-treated HL-60 cells was found by measuring the malondialdehyde (MDA) as previously described [24]. As is aforementioned, the cells were treated with different doses of VAD for 24 h. After treatment, the cells were homogenized and the homogenate was centrifuged at 3000× *g* for 10 min at 4 °C. The supernatants were pooled and 1 mL of this sample was added to a test tube with an equal volume of the solution comprising 20% trichloroacetic acid (TCA) and 0.01% butylated hydroxytoluene (BHT). The samples were heated at 95 °C for 25 min and cooled to room temperature. The absorbance was measured at 532 nm.

### 2.6. Evaluation of DNA Damage by Comet Assay

DNA damage was evaluated using the comet assay as previously described [25,26], with some modifications as also previously described [20]. Briefly, untreated and treated cells with VAD were harvested after 48 h. The cell samples were mixed with 0.5% low-melting-point agarose and pipetted onto a pre-warmed cometslide; then the slides were submerged in pre-chilled lysis solution (1% Triton X-100, 2.5 M NaCl, 1% laurosylsarcosinate and 10 mM EDTA; pH 10.5) for 1 h at 4 °C. After soaking with pre-chilled unwinding and electrophoresis buffer (0.3 M NaOH and 1 mM EDTA; pH 13) for 20 min, the samples were stained with SYBR Green and viewed under an Olympus fluorescence microscope.

### 2.7. Evaluation of Apoptosis and/or Necrosis by Annexin V FITC/PI Assay

To assess whether VAD induces apoptosis and/necrosis in human prostate adenocarcinoma (PC-3) cells, we performed the annexin V FITC/PI assay. Cells were seeded in six-well cell culture plates (Corning Inc., Corning, NY, USA) at a density of 6 × 10^4^ cells/mL and were allowed to grow for 48 h at 37 °C in a 5% CO_2_ incubator. After growing for 2 days, stock VAD extract at a concentration of 100 mg/mL was added directly to the wells in the necessary calculated amounts in order to produce different concentrations of VAD extracts of 125, 250, and 500 µg/mL, which were then incubated for an additional 48 h. After treatment, the cells were harvested and washed with PBS and diluted annexin binding buffer (10 mm Hepes/NaOH, pH 7.4; 140 mm NaCl; 2.5 mm CaCl_2_). The control and treated samples were stained with 5 μL of annexin V FITC and 5 μL of propidium iodide and were incubated for 15 min at room temperature in the dark. The samples were washed with binding buffer and analyzed by the fluorescence-activated cell-sorting (FACS-Vantage) system using the Cell Quest software (Becton-Dickinson, San Jose, CA, USA) within 1 h of staining.

### 2.8. Statistical Analysis

We performed the Student’s paired *t*-test and ANOVA with a subsequent Dunnett’s test using SAS software. All data points are expressed as the mean ± SD of three or more replications, and *p* < 0.05 was considered statistically significant.

## 3. Results

### 3.1. Cytotoxic Efficacy of Vernonia amygdalina Delile on PC-3 Human Prostate Carcinoma Cells

**Figure 1:**
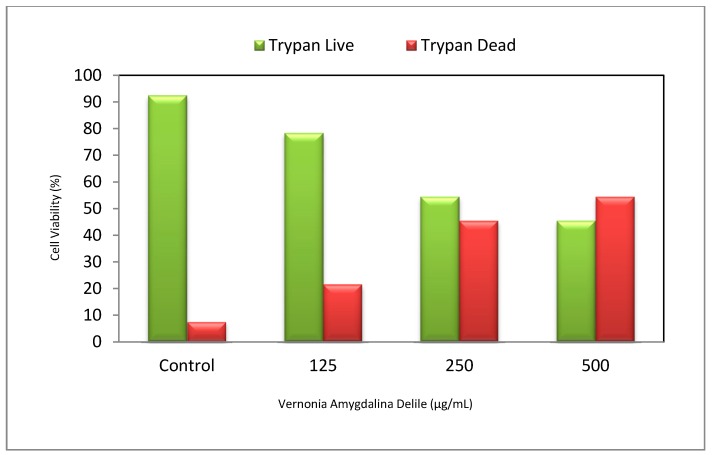
Antiproliferative effect of *Vernonia amygdalina* Delile (VAD) on PC-3 cells. Growth inhibition of PC-3 cells treated with different doses of VAD for 48 h and measured by trypan blue test. Data are means ± SD from three independent determinations in triplicate.

The inhibitory effects of VAD on the growth of PC-3 cells were tested for 48 h using a trypan blue test. Data generated from this test showed that the growth of PC-3 cells was significantly (*p* < 0.05) inhibited, as compared to control cells, in a dose-dependent manner (Figure 1). As seen in Figure 1, VAD doses of 125, 250, and 500 µg/mL inhibited PC-3 cell viability by 14.1%, 38%, and 47% (*p* < 0.05) respectively when compared to the control.

**Figure 2:**
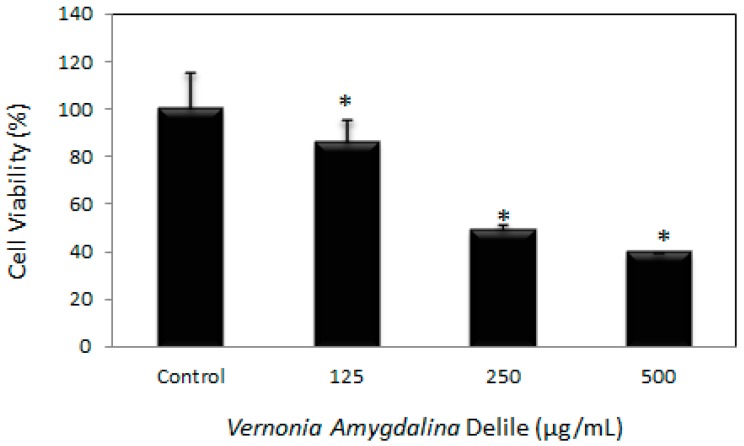
Antiproliferative effect of *Vernonia amygdalina* Delile (VAD) on PC-3 cells. Growth inhibition of PC-3 cells treated with different doses of VAD for 48 h and measured by MTT assay as described in Materials and Methods section. Data are expressed as means ± SD (*n* = 3). * Significantly different from the control by ANOVA and Dunnett’s test; *p* < 0.05.

To confirm that VAD effectively inhibits the growth of cancer cells, the antiproliferative effect of VAD was further determined by a MTT assay, which showed a dose-dependent inhibition of the cell growth (Figure 2). As seen in Figure 2, the IC_50_ value that caused a 50% loss of the PC-3 cell viability was computed to be 196.6 µg/mL upon 48 h of exposure. The present findings demonstrate for the first time that VAD significantly inhibits PC-3 cell growth in a dose-dependent manner, as determined via a trypan blue test (Figure 1) and MTT assay (Figure 2).

### 3.2. Morphological Effects of Vernonia amygdalina Delile on PC-3 Human Prostate Carcinoma Cells

**Figure 3:**
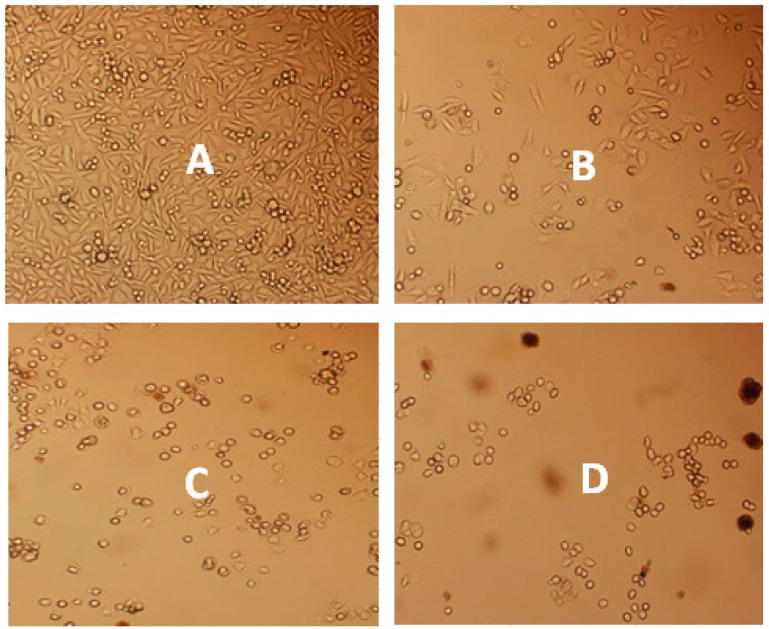
Morphological changes of PC-3 cells were observed under a microscope. The figure shows untreated cells (**A**-control) and cells treated with *Vernonia amygdalina* Delile (VAD) of 125 µg/mL (**B**); 250 µg/mL (**C**); and 500 µg/mL (**D**) for 48 h. Results were confirmed by repeating the experiment three times.

To assess the alterations of cell morphology, PC-3 cells were treated with VAD for 48 h and the morphology of the cells was observed under a brightfield/fluorescent microscope (Figure 3). As seen in Figure 3, the untreated cells (A-Control) retained normal cell morphology and attached firmly to the culture plates with a random orientation. However, the treated cells with 125, 250, and 500 μg/mL of VAD showed remarkable cell damage: decreases in cell number, rounding effects, reduction in cell size, detachment from the substratum, hydropic degeneration of cytoplasm and more apoptotic bodies depending on the dose of VAD. Significant morphological changes were observed for the 500 μg/mL of VAD treatment, presenting features of necrosis such as a loss of membrane integrity, no vesicle formation and complete lysis, as compared to the control cells. These characteristics of cell death were confirmed by Annexin V/PI assay data (Figure 6). This result suggests that VAD has the ability to prevent or inhibit the growth of prostate cancer cells.

### 3.3. Vernonia amygdalina Delile Modulated Oxidative Stress on PC-3 Human Prostate Carcinoma Cells 

**Figure 4:**
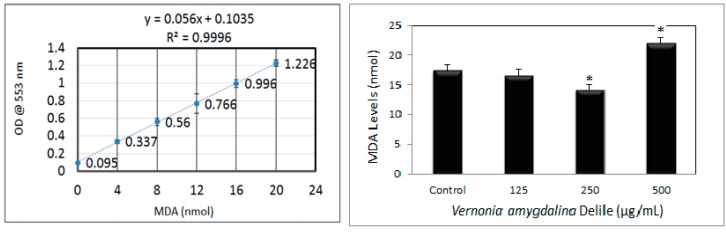
Malondialdehyde (MDA) standard curve (**Left**) and effects of *Vernonia amygdalina* Delile (VAD) extract on MDA level in PC-3 cells (**Right**). PC-3 cells were treated with various doses of VAD: 125, 250 and 500 µg/mL. MDA concentrations were determined as described in Materials and Methods section. Data are expressed as means ± SD (*n* = 3). * Significantly different from the control by ANOVA and Dunnett’s test; *p* < 0.05.

Lipid peroxidation was estimated by measuring the MDA formation, and the results showed that VAD gradually decreases MDA generation in PC-3 cells treated at 125 and 250 µg/mL, likely due to its antioxidant properties (Figure 4). However, a significant (*p* < 0.05) increase in the MDA level was observed with 500 µg/mL of VAD, in comparison to the control group, perhaps due to a high level of cell death at the higher dose of exposure. This finding suggests that VAD acts as a good antioxidant and inhibits lipid peroxidation in PC-3 cells below the IC_50_ value, but also acts as pro-oxidant at the higher dose of exposure above the IC_50_ value (Figure 4).

### 3.4. Genotoxic Effects of Vernonia amygdalina Delile on PC-3 Human Prostate Carcinoma Cells 

**Figure 5:**
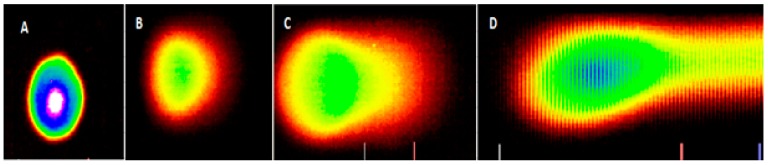
Representative SYBR Green comet assay images of untreated control (**A**) and *Vernonia amygdalina* Delile (VAD)-treated PC-3 cells at 125 µg/mL (**B**); 250 µg/mL (**C**); and 500 µg/mL (**D**). Cells observed in the treated images for each VAD dose exhibited an increase in DNA damage as VAD dose rose.

The comet assay is a straightforward method to assess DNA strand breaks in eukaryotic cells, and the methodology is relatively simple. By means of the comet assay, we elucidated in the present study some of the molecular changes in PC-3 cells treated with VAD (Figure 5). Our results demonstrated that VAD induced DNA damage in PC-3 cells in a dose-dependent fashion, giving clear evidence that VAD may be a potent DNA-damaging anticancer agent effective against prostate cancer. As seen in Figure 5, there is a significant difference in the distance of the migration of the DNA fragments, as well the intensity of the fragments’ localization. A higher dose of VAD was revealed to cause the most damage; hence, the higher intensity of the fragments’ migration from the head region of the comet to the tail region. With the lowest treatment dose, there was no migration, suggesting intact DNA without damage.

### 3.5. Apoptotic and Necrotic Effects of Vernonia amygdalina Delile on PC-3 Human Prostate Carcinoma Cells 

**Figure 6:**
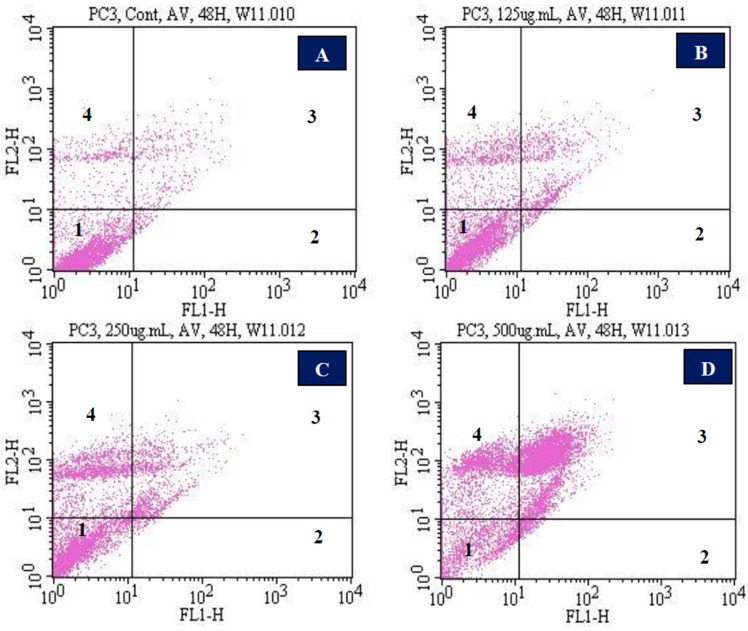
Representative dot plots showing the apoptotic and necrotic potential of *Vernonia amygdalina* Delile (VAD) in PC-3 cells upon 48 h of extract exposure. (**A**): Controls (untreated); (**B**): 125 µg/mL VAD; (**C**): 250 µg/mL VAD; (**D**): 500 µg/mL VAD. Quadrant 1: Live cells or annexin V- and PI-negative cells; 2: Early apoptotic or annexin V-positive cells; 3: Late apoptotic and necrotic or annexin V- and PI-positive cells; 4: Necrotic or PI-positive cells.

To evaluate whether VAD induced apoptosis- and/or necrosis-mediated cell death, we used annexin V and PI double staining to detect cell membrane changes. Figure 6 shows representative flow cytometry dot plots of both the untreated and treated PC-3 cells for 48 h. We observed that VAD caused a dose-dependent increase in the number of PC-3 cells, expressing both stages of apoptosis as well as necrosis. Viable cells were negative for both annexin V and PI (quadrant 1); early apoptotic cells were positive for annexin V and negative for PI (quadrant 2), whereas late apoptotic or necrotic cells displayed both high annexin V and PI responses (quadrant 3); and non-viable cells undergoing necrosis were positive for PI and negative for annexin V (quadrant 4). The percentages of both annexin V- and PI-positive cells were (9.1 ± 0.212)%, (18.8 ± 1.004)%, (29.5 ± 2.86)%, and (82.1 ± 0.800)% for 0, 125, 250, and 500 µg/mL doses, respectively (Table 1).

## 4. Discussion

### 4.1. Cytotoxic Efficacy of Vernonia amygdalina Delile on PC-3 Human Prostate Carcinoma Cells 

To evaluate the effectiveness potential of VAD on PC-3 cells and to determine its anticancer activity potential, we performed the trypan blue test, MTT assay, and cell morphology analysis. Data generated from these series of experiments indicated that VAD remarkably inhibits the growth of PC-3 cells in a dose-dependent fashion. From the cell morphology result (Figure 3), we observed that tumor cells were spreading and proliferating in the control group. However, the treated group revealed a sign of tumor cell shrinkage and a clustering tendency. Consistent with our findings, previous studies demonstrated that phytochemical extracts of medicinal herbs exhibit anticancer activities and are valuable natural sources for drug-like active natural compound screenings [27,28,29,30]. Working with breast cancer (MCF-7) cells, previously published data in our lab showed that *V. amygdalina* treatment reduces cellular viability and induces DNA damage leading to apoptosis accompanied by secondary necrotic cell death in tumor cells [20,21]. A 2012 report indicated that the natural medicinal plant works with the body to boost the immune system by killing unhealthy cells [31]. In 2015, our research group used diverse medicinal plants to treat 328 Cameroonian patients who had been diagnosed with diabetics and/or hypertension by a physician or medical professional for a period of 10 days. At the end of the 10 day treatment, we found that 70% of the patients had a complete remission and were free from diabetes and/or hypertension [32].

In France and Germany, many plant extracts are used as prescribed medicines [33]. The number of medicals tending to use such herbal medicines is increasing [34]; patients who are suffering from cancer especially are inclined to use herbal medicine in the hope to cure or improve the disease, prevent the disease from converting to metastatic form, support the immune system, reduce stress, and relax [34]. More importantly, the main reasons for using herbal medicine in cancer treatment are: (1) the prevention of cancer by creating an unfavorable environment for the growth of cancer cells, (2) the prevention of a recurrence of cancer, (3) to increase the body’s immune system, and (4) to reduce side effects resulting from using modern treatment methods such as chemotherapy and radiotherapy [35,36].

### 4.2. Vernonia amygdalina Delile-Modulated Oxidative Stress on PC-3 Human Prostate Carcinoma Cells

The ability of VAD to modulate oxidative stress was estimated by measuring the level of lipid peroxidation products in PC-3 cells. The results showed that VAD modulates oxidative stress by decreasing the production of MDA levels in PC-3 cells treated with 125 and 250 µg/mL, likely because of its antioxidant properties (Figure 4). However, a significant (*p* < 0.05) increase in the MDA level was observed for 500 µg/mL of VAD in comparison to the control group, likely as a result of a high level of cell death at the higher dose of exposure, as revealed by the MTT assay. This finding suggests that VAD is a good antioxidant and inhibits lipid peroxidation when the cell treatment is below the IC_50_ value, but also acts as a pro-oxidant at a higher dose of exposure when the cell treatment is above the IC_50_ value (Figure 4). Studies indicated that in some cases, high concentrations of antioxidants may have pro-oxidant activity. One example is ascorbic acid, which is well-known to act as a pro-oxidant at very high concentrations [37]. Antioxidants are natural substances that may prevent or delay some types of cell damage. Fruits and vegetables are great sources of antioxidants [38]. In fact, an antioxidant is a redox agent that may become a pro-oxidant to accelerate lipid peroxidation and induce DNA damage under special conditions and concentrations [39]. Scientific reports have revealed the pro-oxidant effects of antioxidant vitamins and many classes of plant-derived polyphenols, including curcumin [40], flavonoids [41], and tannins [42]. Among the many factors that cause cancer, oxidative stress is one of the key important and well-studied events that give rise to the conditions leading to tumor onset and progression [43].

### 4.3. Genotoxic Effects of Vernonia amygdalina Delile on PC-3 Human Prostate Carcinoma Cells

Single-cell gel electrophoresis, also known as a comet assay, was used to evaluate the degree of DNA damage [44,45]. This method uses an electric field to resolve negatively charged DNA fragments in an agarose gel. The migration of DNA fragments is directly proportional to the amount of DNA damage in the cell. Using the comet assay, we demonstrated that VAD significantly induced DNA strand breaks in PC-3 cells compared to the control in a dose-dependent fashion (Figure 5). A higher dose of VAD was revealed to have caused the most DNA damage, and hence the higher intensity of the fragments’ migration from the head region of the comet to the tail region. With the lowest treatment dose there was no migration, suggesting intact DNA without damage. A previous report from our lab indicated that HL-60 cells treated with different concentrations of arsenic trioxide led to the inhibition of cell growth, and the induction of DNA damage and apoptosis associated with oxidative stress [46,47]. To the best of our knowledge, no data is found in the literature regarding the genotoxic effect of VAD in cell or animal models. Here, we document for the first time that VAD may represent a DNA-damaging anticancer agent effective against prostate cancer.

### 4.4. Apoptotic and Necrotic Effects of Vernonia amygdalina Delile on PC-3 Human Prostate Carcinoma Cells 

Cancer cells evolve to avoid the apoptosis-inducing signaling pathway in order to survive [48]. Therefore, the induction of apoptosis in tumor cells can be an effective treatment approach for cancer therapy. Medicinal plants, regardless of crude extracts or isolated active ingredients, have drawn increasing attention as anticancer agents because of their ability to trigger apoptosis [49,50,51]. Given the effectiveness of VAD on the suppression of cell growth and induction of DNA damage, we tested the apoptotic and necrotic effects of VAD on PC-3 cells. Treatment with VAD significantly induced both cell apoptosis and necrosis of PC-3 cells in a dose-dependent manner. In agreement with our findings, a previous study demonstrated that in response to a given death stimulus, there was often a continuum of apoptosis and necrosis (Majno and Joris 1995) [52]. This study by Majno and Joris revealed that many insults induce apoptosis at lower doses and induce necrosis at higher doses. Even in response to a certain dose of a death-inducing agent, features of both apoptosis and necrosis may coexist in the same cell. Dead cells in the late stages of apoptosis may present necrotic features, as a result of the loss of cellular energy and plasma membrane integrity (Majno and Joris 1995) [52]. This process is called “apoptotic necrosis” or “secondary necrosis”.

The induction of both apoptosis and necrosis in PC-3 cells by VAD may be the important mechanism of its action against prostate cancer. The ability of VAD to induce cell death using staining with annexin V FITC/PI confirms that its underlying mechanism of antiproliferation is mainly mediated by an induction of apoptosis and necrosis. However, further studies are required to establish the molecular mechanism behind the antiproliferative effect of VAD on prostate cancer cells. Apoptosis induction is one of the main applications of chemopreventive plants [53,54]. The above results are similar to an earlier report that *V. amygdalina*-induced growth arrest and apoptosis are accompanied by secondary necrosis of human breast adenocarcinoma (MCF-7) cells [21]. It has been shown that many natural plants induce apoptotic pathways that are blocked in cancer cells. For example, genistein has been shown to induce apoptosis in human promyelocytic HL-60 leukaemic cells [55]; biocalein (a flavenoid contained in sho-saiko-to herbal medicine) has been shown to induce apoptosis in human hepatocellular carcinoma cells [55]; ginger has been shown to induce cytotoxic activity in cancer cells through apoptosis [51]; and curcumin has been shown to cause cell proliferation arrest and induce apoptosis in several types of human and animal cells, including gastric and colon cancer cells [56], and human melanoma cells [57].

## 5. Conclusions

Patients who are suffering from cancer are inclined to use medicinal plants in the hope to cure and improve the disease, prevent the disease from converting to metastatic form, support the immune system, reduce stress, and relax [30]. Medicinal plants have traditionally played an important role in the socio-cultural, spiritual and health arena of rural and tribal areas around the world. In the present study, we tested the therapeutic efficacy of VAD and established its antiproliferative effects against prostate cancer. We showed that VAD causes cell death through the induction of cell growth arrest, oxidative stress, DNA damage, apoptosis, and secondary necrosis in vitro. These results provide useful data on the anticancer activities of VAD in prostate cancer and demonstrate the novel possibilities of this medicinal plant for developing prostate cancer therapies. Our study is the first to demonstrate the in vitro therapeutic effect of VAD on human prostate carcinoma cells. However, its anticancer activities in vivo and the mechanisms of action are not completely understood. Currently, we are attempting to identify VAD’s biologically active ingredient. In a future study, we will test the chemotherapeutic effects of VAD’s biologically active ingredient and underlie its therapeutic mechanisms.

## Figures and Tables

**Table 1:** Summary of annexin V/PI data obtained from flow cytometry (Figure 6). Human prostate adenocarcinoma (PC-3) cells were cultured in the absence or presence of VAD for 48 h as described in the Materials and Methods section. Values are shown as the means ± SD of three replicates per experiment.

Percentage of Cells and Corresponding Responses in Annexin V/PI Assay		
Doses (µg/mL VAD)	Annexin V and PI Negative or Viable Cells (Mean ± SD) %	Annexin V and PI Positive Cells (Mean ± SD) %
Control	90.9 ± 0.212	9.1 ± 0.212
125	81.2 ± 1.0 *	18.8 ± 1.0 *
250	70.5 ± 2.9 *	29.5 ± 2.9 *
500	17.9 ± 0.8 *	82.1 ± 0.8 *

* *p* < 0.05 compared with control group.
